# Liquid Crystal Formation from Sunflower Oil: Long Term Stability Studies

**DOI:** 10.3390/molecules21060680

**Published:** 2016-06-09

**Authors:** Pedro Alves da Rocha-Filho, Mônica Maruno, Márcio Ferrari, José Fernando Topan

**Affiliations:** 1 Department of Pharmaceutical Sciences, Faculty of Pharmaceutical Sciences of Ribeirão Preto, University of São Paulo, Avenida do Café, s/n. Bairro Monte Alegre, Ribeirão Preto, SP 14040-903, Brazil; topan@fcfrp.usp.br; 2 Centro Universitário Barão de Mauá, R. Ramos de Azevedo, 423, Jardim Paulista, Ribeirão Preto, SP 14090-180, Brazil; monica.maruno@baraodemaua.br; 3 College of Pharmacy, Federal University of Rio Grande do Norte, Rua Gustavo Cordeiro de Farias, s/n, Petrópolis, Natal 59012-570, RN, Brazil; ferrarimarcio@uol.com.br

**Keywords:** sunflower oil, liquid crystal, fatty esters, cosmetic products

## Abstract

The Brazilian biodiversity offers a multiplicity of raw materials with great potential in cosmetics industry applications. Some vegetable oils and fatty esters increase skin hydration by occlusivity, keeping the skin hydrated and with a shiny appearance. Sunflower (*Helianthus annus* L.) oil is widely employed in cosmetic emulsions in the form of soaps, creams, moisturizers and skin cleansers due to the presence of polyphenols and its high vitamin E content. Liquid crystals are systems with many applications in both pharmaceutical and cosmetic formulations and are easily detected by microscopy under polarized light due to their birefringence properties. The aim of this research was to develop emulsions from natural sunflower oil for topical uses. Sunflower oil (75.0% *w*/*w*) was combined with liquid vaseline (25.0% *w*/*w*) employing a natural self-emulsifying base (SEB) derivative. The high temperature of the emulsification process did not influence the antioxidant properties of sunflower oil. Fatty esters were added to cosmetic formulations and extended stability tests were performed to characterize the emulsions. Fatty esters like cetyl palmitate and cetyl ester increase the formation of anisotropic structures. O/W emulsions showed acidic pH values and pseudoplastic behavior. The presence of a lamellar phase was observed after a period of 90 days under different storage conditions.

## 1. Introduction

Liquid crystals (LCs) are self-assembled dynamic functional soft materials which possess both order and mobility at the molecular, supramolecular and macroscopic levels. For this reason LCs are referred to as intermediate phases or mesophases and called the fourth state of matter. Liquid crystalline structures have received a good deal of attention in recent years. The liquid crystalline structures formed by amphiphilic molecules form the basis for emulsions and have been thoroughly studied by researchers in cosmetic product development. Many cosmetic products like skin lotions and creams are based on water being the solvent and some of these products have in common the presence of liquid-crystalline phases which influence the rheological properties. Liquid crystals are even more promising because of their important role in biology. In a more general sense, the combination of order and mobility, as exhibited by liquid crystals, is a basic requirement for self-organization and structure formation in living systems [1]. So far liquid crystalline structures have been discussed as affecting the structure and stability of oil and/or water. Knowledge of the structural and chemical composition of the human epidermis can be useful in cosmetic products. The repair of the lamellar structures in the epidermis or the supply of essential fatty acids or ceramides should result in a well-developed skin barrier. During cell differentiation, from the stratum basal of the epidermis to the upper part—the horny layer—the chemical composition of skin lipids changes and between them liquid crystalline structures fuse to form lamellar lipid membranes of fatty acids, cholesterol and ceramides which together determine the properties of this water barrier. Hence, the reduction in transepidermal water loss increases the water content of the upper skin and wrinkle formation should be significantly reduced [2].

Liquid crystals are systems that can be found in many emulsions with many applications in both pharmaceutical and cosmetic formulations and are easily detected by microscopy under polarized light due to their birefringence property [3].Santos *et al.* [4] have found liquid crystal in cosmetic emulsions employing calendula oil. Sunflower oil contains various kinds of fatty acids: saturated (lauryl: C12:0, myristic C14:0; palmitic C16:0, and stearic C18:0), monounsaturated (oleic acid C18:1, n-9), and polyunsaturated (linoleic acid C18:2, n-6, and α-linolenic C18:3, n-3) (Pedersen, 2000) [5].

Sunflower oil is widely used in cosmetic emulsions like soaps, creams, moisturizers and skin cleansers due to the presence of polyphenols and its high vitamin E content [6], which is very important in cosmetic products due to its antioxidant properties [7,8]. The aim of this research was to develop emulsions from sunflower natural oil and natural vegetable butter for topical uses. Sunflower oil (75.0% *w*/*w*) was combined with liquid vaseline (25.0% *w*/*w*) employing a natural self-emulsifying base (SEB) derivative. The influence of fatty esters on the cosmetic formulation and extended stability tests were performed and the presence of lamellar phases was evaluated for a period of 90 days under different storage conditions.

## 2. Results and Discussion 

### In Vitro Determination of the Antioxidant Activity of Sunflower Oil by H^+^ Donor Activity to Radical DPPH^•^

**Figure 1:**
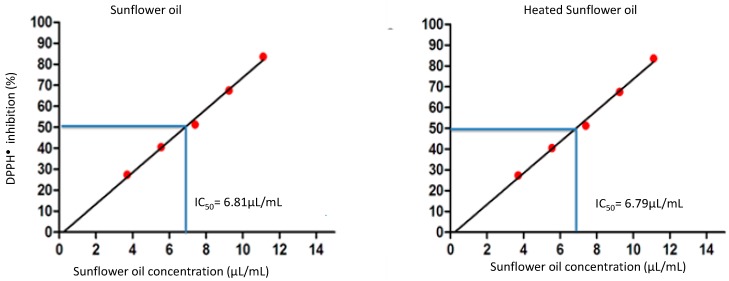
Determination of the activity of H^+^ donor of sunflower oil and sunflower oil heated (75 ± 2 °C) to the DPPH^•^.

According to Bandoniene [9] the IC_50_ value expresses the amount of antioxidant raw material needed to decrease by 50% the absorbance of DPPH^•^, namely to cause a 50% DPPH^•^ inhibition. Sunflower oil showed an IC_50_ value of 6.81 µL/mL, meaning that 6.81 µL/mL of sunflower oil inhibits the activity of the DPPH^•^ by 50%, while the IC_50_ value of the sunflower oil after heating is 6.79 µL/mL (Figure 1). Comparing the H^+^ donor activity of sunflower oil and heated sunflower oil, no statistically significant difference (*p* < 0.05) in this activity was found, thus suggesting that the high temperature used for the emulsification process does not influence the antioxidant properties of sunflower oil. From this test we can assume that sunflower oil may be heated at the emulsification temperature while maintaining its antioxidant properties.

**Figure 2:**
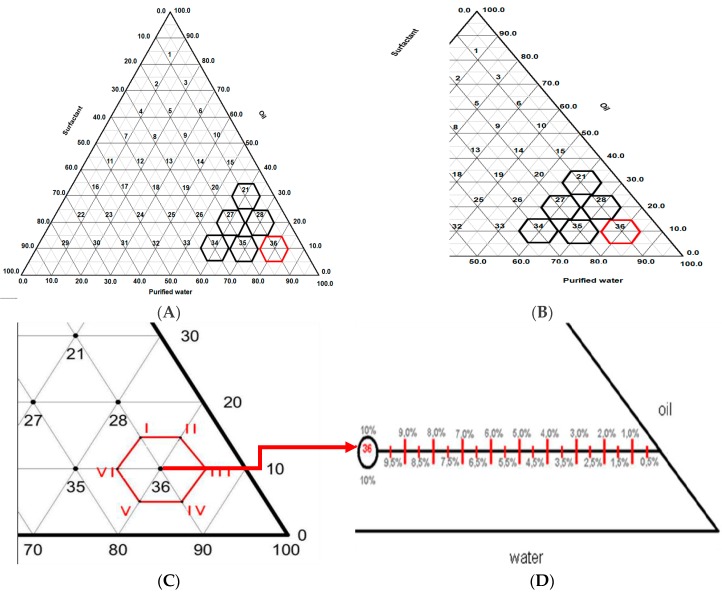
Derivatives of the formula 36 (**A**) pseudo-ternary diagram; (**B**) details of the pseudo-ternary diagram showing points 21, 27, 28, 34, 35 and 36; (**C**) derivative formulas around point 36; (**D**) maintaining the oil concentration, varying the water and decreasing the concentration of surfactant.

Figure 2A shows a pseudo-ternary phase diagram and 2B some formulas (21, 27, 28, 34, 35 and 36) with high water content. According to Marti-Mestres [10] O/W emulsions have better spreadability, are easily absorbed and leave no oily feeling on the skin. Aiming to develop O/W emulsions only the region of the ternary diagram with higher water concentration (Figure 2C) was used. Formula 36 in the ternary diagram composed of oils (sunflower 75.0% and 25.0% mineral oil), surfactants (75.0% of SEB I and 25.0% SEB II) and purified water showed a lamellar liquid crystalline phase. After choosing the best concentration of oil phase and surfactant—point (36) of the ternary diagram—the pseudo-ternary diagram was made varying the component concentrations by 5.0% *w*/*w* (Figure 2C), resulting in the six new formulations described in Table 1.

After obtaining formulations derived from point 36 in the pseudo-ternary diagram, formulation III was chosen and it was observed that stable emulsions were obtained with liquid crystals even if the concentration of surfactants was decreased. Oil concentration was maintained, varying the water and decreasing the concentration of surfactant, which means a decrease in the surfactant concentration in the formula (Figure 2D).

**Figure 3:**
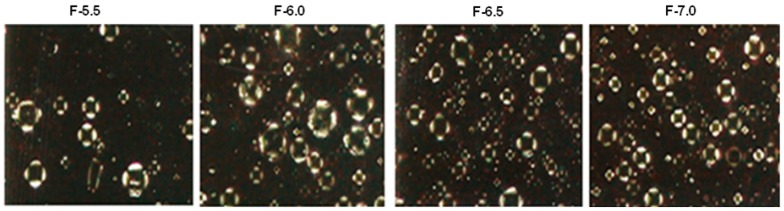
Polarized light photomicrographs (200×) of the formulations after 24 h.

The formulations (Table 2) with lower concentrations of surfactant (<3.5%) showed signs of instability 24 h after preparation along with coalescence and creaming. The remaining formulations (4.0 to 10.0) were stable and only the formulations 5.5; 6.0; 6.5; 7.0 (Table 3) evaluated macroscopically and microscopically under polarized light showed homogeneous liquid crystals (Figure 3) and were selected for further preliminary stability test studies.

The long-term behavior of the emulsions can be estimated by centrifugation at moderate speeds. According to ANVISA [11] primary stability studies are not intended to estimate the lifetime of the product but rather help in the screening of formulations. The products subjected to centrifugation tests at a high gravity force undergo coalescence processes that do not occur under normal gravity conditions. However, from the moment that it is subjected to a very high gravity force, if the system does not suffer changes, it can be said that the emulsion would be stable for a long period of time. As it can be seen in the Table 3 the formulations showed no phase separation during the centrifugation tests.

According to Latreille *et al.* [12] the stability is directly proportional to the force of gravity, so the long-term behavior of emulsions can be estimated by centrifugation at moderate speeds. However, the centrifugation stability test is only indicative, but must be combined with other tests such as thermal stress and accelerated stability [13]. Some formulations remained homogeneous after the thermal stress test, being stable up to 60 °C with slight modifications at 70 °C.

The pH values of emulsions had acidic character, perhaps associated with the acidic character of the SEB I surfactant, with a pH value around 3.1 [14]. The electrical conductivity values indicate the hydrophilic character of the external phase confirming the O/W emulsion type.

Prolonged stability tests of formulations under different storage conditions may indicate long-term physical stability and consistency changes as a function of time [13], so the emulsions were subjected to prolonged stability tests under certain storage conditions for a period of 90 days [15]. Under the different storage conditions among the selected formulations (5.5, 6.0, 6.5 and 7.0) formulations 5.5 and 6.0 showed signs of instability such as creaming and coalescence from the 60th day.

Only for formula 6.5 at 40 ± 2 °C was a statistically significant difference observed on the thirtieth day for the pH value (3.45 ± 0.15). The remaining samples maintained the pH value with minimal variation within 90 days of analysis.

Comparing the studied formulations, pH values presented acidic characteristics (3.5 ± 0.1) and for the formulations 6.5 and 7.0 maintained at 25 ± 2 °C and 5 ± 2 °C there was slight variation during the accelerated stability test while the formulations stored at 40 ± 2 °C showed statistically significant variations (3.4 ± 0.2). This decrease in pH may be due to hydrolysis of fatty acid esters present in oils, leading to the generation of free fatty acids reducing the pH value [16]. These results are in agreement with Andrade *et al.* [17] who used the same self-emulsifying phosphoric ester base and andiroba oil demonstrating the nature of the acidic phosphoric base. 

The electrical conductivity values (220.00 ± 20.0 µS/cm) initially were maintained and at 40 ± 2.0 °C an increase of this value (270.00 ± 50.0 µS/cm) was observed This increase in electrical conductivity of formulations subjected to storage temperature of 40 ± 2.0 °C can be related to coalescence. 

According to Andrade *et al.* [17] formulations stored at high temperatures tend to decrease in consistency and their free water content increases, causing an increase in the electrical conductivity of the system. Masmoudi *et al.* [16] stated that generally when an emulsion was stable the conductivity values were unchanged. However, these authors also reported stable emulsions stored at 50 °C for which the conductivity value increased although no destabilization process was observed in macroscopic analyses. Our results were similar because the formulation samples stored at 40 ± 2.0 °C also showed no signs of instability. Masmoudi *et al.* [16] also stated that emulsions that have high electrical conductivity values tend to be more unstable, and this is in agreement with our results. For other storage conditions (5 ± 2.0 °C and 25 ± 2.0 °C) the conductivity values remained constant and no statistically significant changes were observed. 

**Figure 4:**
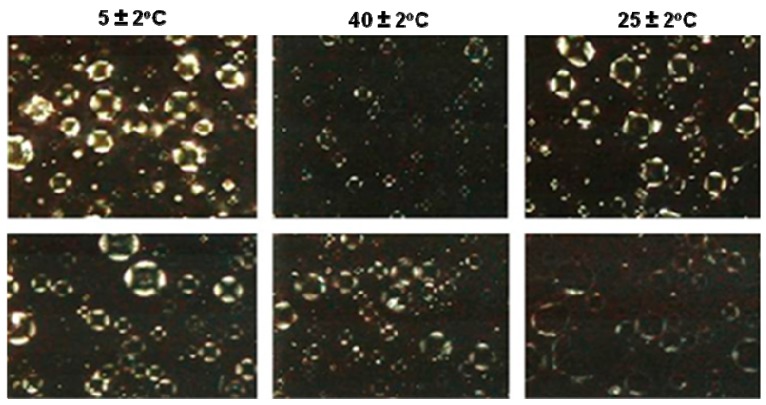
Polarized light photomicrograph (200×) of formula 6.5 after 24 h and 90 days under various storage conditions.

**Figure 5:**
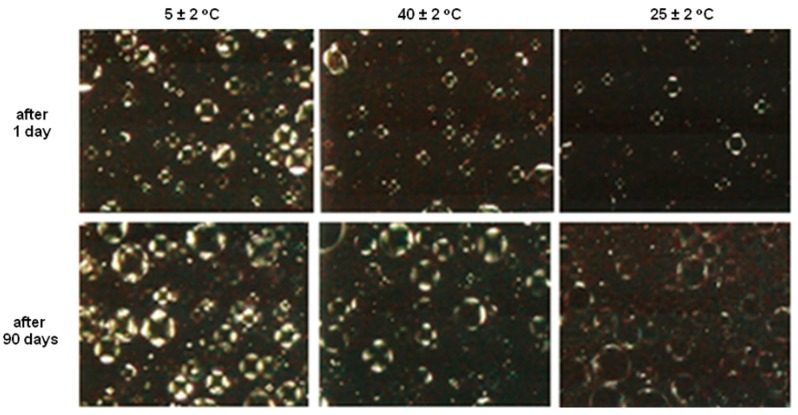
Polarized light photomicrograph (200×) of formula 7.0 after 24 h and 90 days under various storage conditions.

The presence of lamellar structures even after storage at elevated temperature (40 ± 2 °C) may be explained by the fact that these structures are capable of storing free water between their lamellae, a process that occurs during the preparation of the formulation and is maintained throughout the storage period [15]. Lamellar structures have higher moisturizing power compared to formulations without liquid crystal and this increased moisture is caused by free water stored between the lamellae [18]. In photomicrographs it was possible to observe the presence of anisotropic lamellar structures in all formulations within 90 days under extreme storage conditions (Figure 4—formula 6.5 and Figure 5—formula 7.0, respectively).

Straight and branched fatty esters chains are widely used in cosmetic products. The 7.0 formulation was selected to study the influence of the separate addition of fatty esters (1.0%) and to observe the maintenance of liquid crystals.

In agreement with Eccleston [17] and Santos *et al.* [19] the formation of liquid crystal depends on the interaction of amphiphilic surfactant molecules and water. When the surfactant and water are subjected to a temperature increase, the lipophilic chains rearrange themselves into a disordered liquid and the water penetrates between the hydrophilic chains. After cooling, the hydrophilic chains reorganize themselves and retain water between them, thus forming the liquid crystal phase or gel. There is probably an interaction between the carbon chains of surfactants with the carbon chains of the esters (alkyl benzoate, sodium lauryl lactate, benzyl myristate and PPG-3 benzyl ether myristate) used in the emulsification process and when the system is cooled the carbon chains of the surfactants were reorganized, but did not maintain the water between the lamellae and thus do not form liquid crystals.

The formulations added separately with other fatty esters—cetyl lactate, cetyl palmitate, isopropyl myristate, capryl caprylic triglicerides, octyl stearate and esters cetyl esters (1.0%)—were subjected to preliminary and accelerated stability tests and pH, electrical conductivity, and rheological behavior were determined after being subjected to extreme storage temperatures to evaluate the influence of the fatty esters on the formulation insofar as the maintenance of liquid crystals is concerned.

The pH values of the formulations with fatty esters showed statistically significant differences for all fatty esters tested. Formulations containing cetyl ester (3.3 ± 0.10) and isopropyl myristate (3.5 ± 0.10) showed more homogeneous behavior when compared with the other esters. Comparing both formulations with fatty esters—formula 7.0 and without additives—similar pH values were determined, which allows us to presume that fatty esters do not influence the pH values of the formulations.

Regarding the study of electrical conductivity, all formulations show a decrease in electrical conductivity values till 30 days (230.00 ± 20.0 µS/cm). According to ANVISA [11] the increase in conductivity can be taken as a sign of system instability, what was not observed macroscopically in these formulas. The esters cetyl palmitate and cetyl ester showed less electrical conductivity variation during the stability tests. Besides improving emulsion stability, the fatty esters cause a decrease in conductivity value compared with formula 7.0.

**Figure 6:**
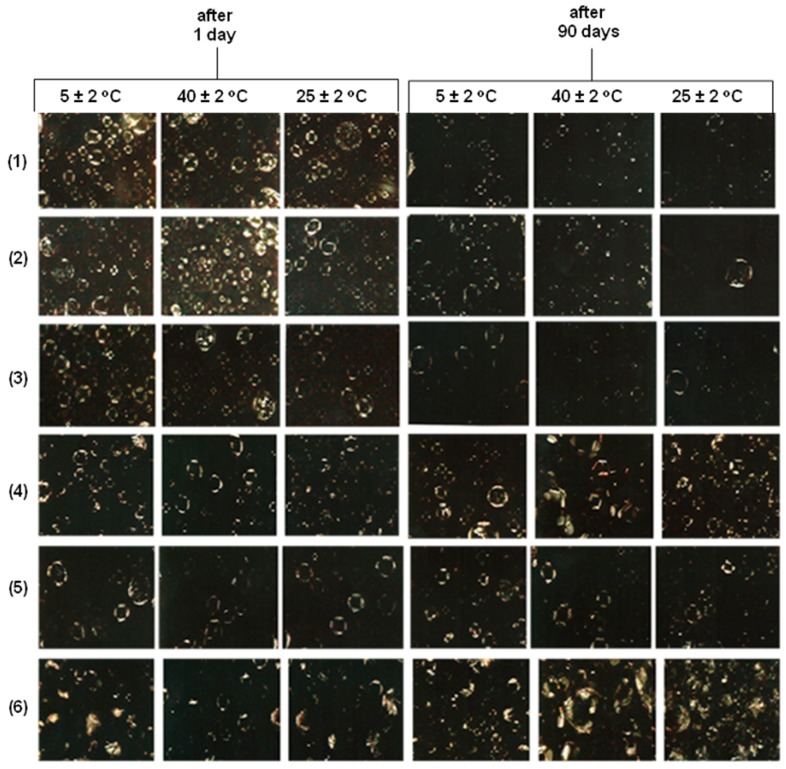
Polarized light photomicrographs (200×) of the formulas doped with 1.0% ester: (**1**) Isopropyl Myristate; (**2**) Cetyl Lactate; (**3**) Capric Caprylic Triglycerides; (**4**) Cetyl Esters; (**5**) Octyl Stearate; (**6**) Cetyl Palmitate) stored under different conditions during the Prolonged Stability Test.

The formulations containing cetyl lactate, caprylic triglycerides, isopropyl myristate and octyl stearate fatty ester showed a decrease in lamellar structures at a storage temperature of 25 ± 2 °C, while formulas containing cetyl palmitate and cetyl ester fatty esters showed increased presence of anisotropic structures. The presence of anisotropic structures was observed in all formulations and these liquid crystal structures remained after periods over 90 days (Figure 6).

**Figure 7:**
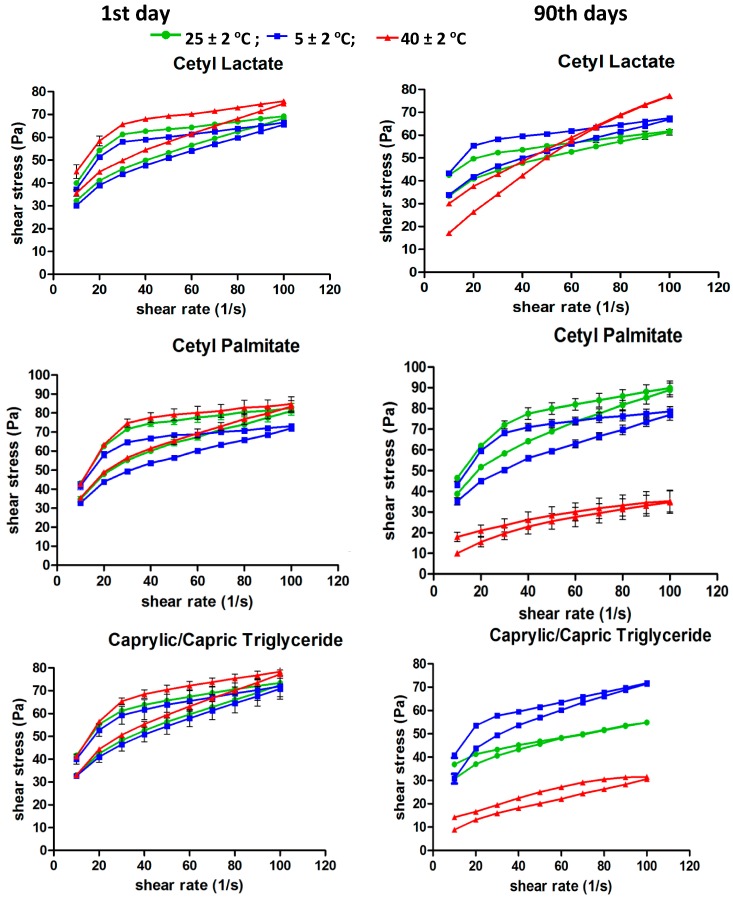

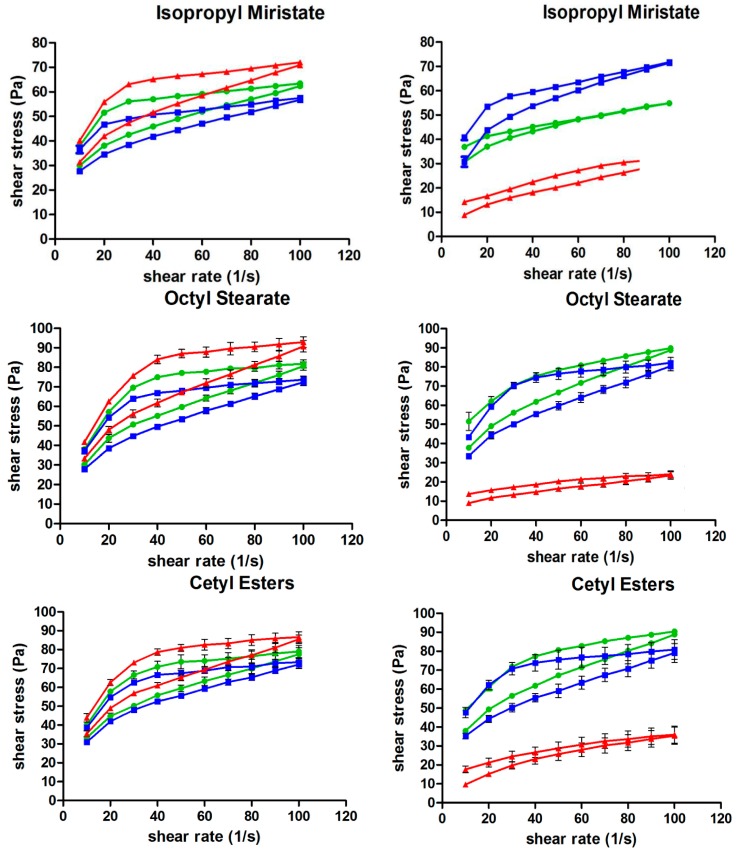
Behavior of emulsions subjected to different stored conditions according to time (*n* = 3).

The rheological behavior of the formulations with additive were evaluated to analyze the influence of fatty esters on the rheological profile during the accelerated stability test. According to the rheograms all samples doped with fatty esters showed thixotropic formulation characteristics that are ideal for cosmetic formulations. We observed a reduction of shear stress and hysteresis area for the formulations stored at room temperature 40 ± 2 °C for a period of 90 days, however, no signs of instability such as coalescence or creaming were observed. This reduction in the hysteresis area is due to the formulation stress when subjected to high temperatures for a long period of time. In Figure 7 we can observe the viscosity of emulsions subjected to different storage conditions according to time.

**Figure 8:**
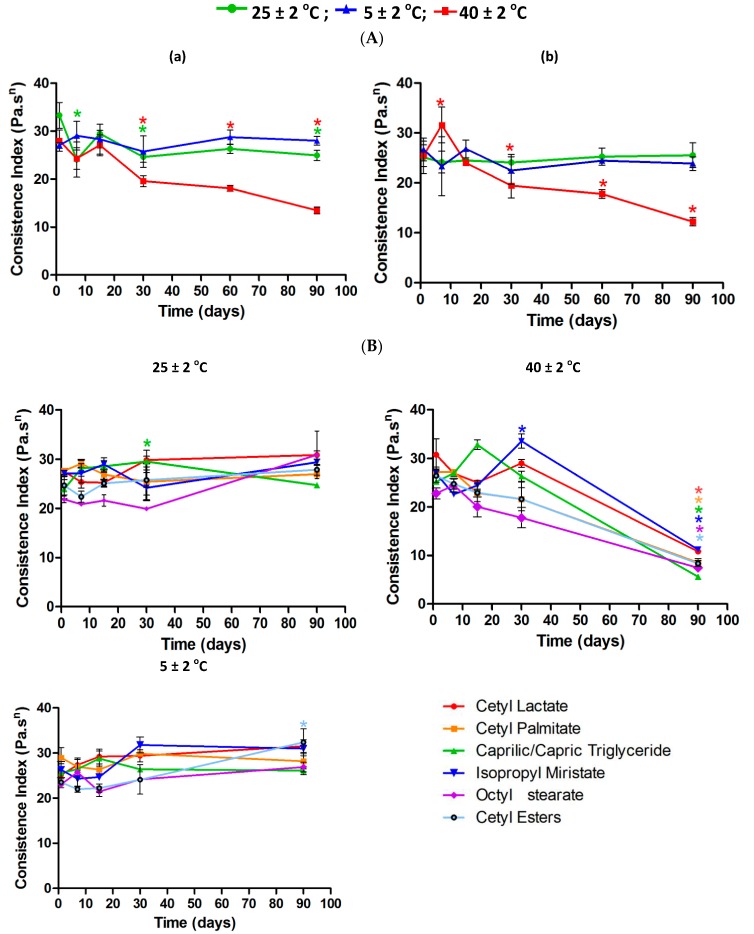
Consistency index for the 6.5 (**a**) and 7.0 (**b**) formulations during Prolonged Stability Test (*n* = 3) without (**A**) or with (**B**) fatty esters.

The consistency index was constant for formulations 6.5 and 7.0 stored at 5 ± 2 °C and 25 ± 2 °C, indicating that the formulations were stable (Figure 8).

At 40 ± 2 °C the formulations showed statistically significant changes compared to zero (t_0_) time. Andrade *et al.* [17] also verified that the consistency index decreased as a function of high temperatures. Pseudoplasty can be observed in the rheograms as the characteristic flow rate value. The flow rate of a formulation is indicative of flow behavior: when it is equal to 1.0, the composition shows Newtonian flow behavior; values below 1.0 correspond to pseudoplastic behavior, which increases with decreasing value of flow rate and values above 1.0 indicate dilatant behavior [19].

**Figure 9:**
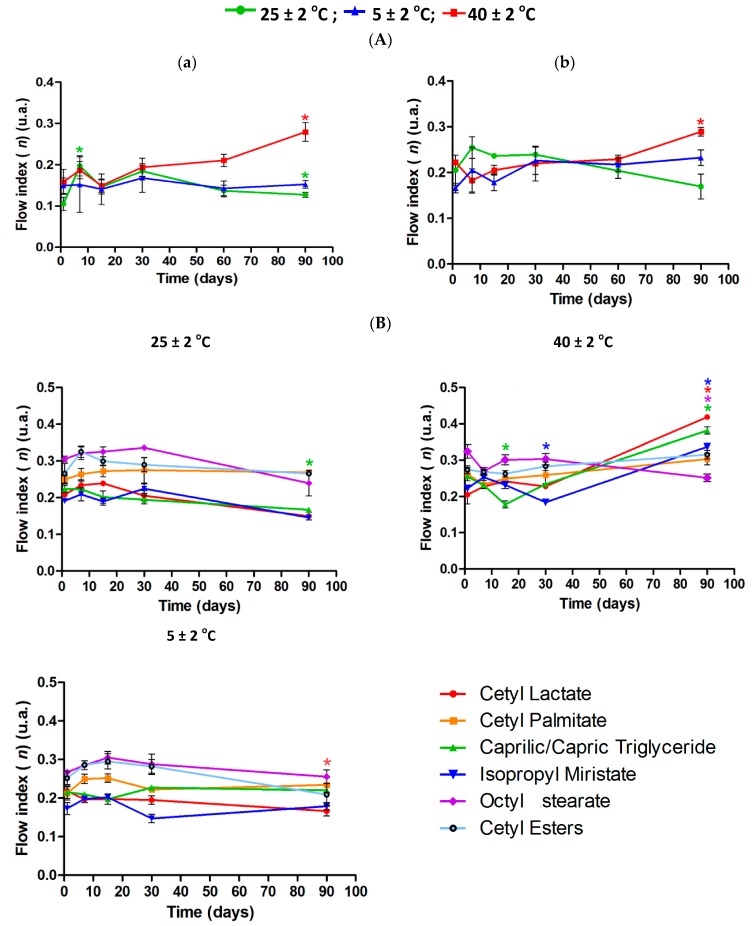
Flow index for 6.5 (**a**) and 7.0 (**b**) formulations during Prolonged Stability Test (*n* = 3) without (**A**) or with (**B**) fatty esters.

Regarding the flow indexes of formulations stored at 5 ± 2 °C, they were stable throughout the test, while the formulations kept at 25 ± 2 °C and 40 ± 2 °C showed statistically significant changes, and the formulations kept at 40 ± 2 °C showed an increase in the flow index (Figure 9), but the value remained below 1.0, as typical of pseudoplastic bevahior. These results were also similar to those of Andrade *et al.* (2008) [17] with cosmetic O/W emulsions containing liquid crystals.

**Figure 10:**
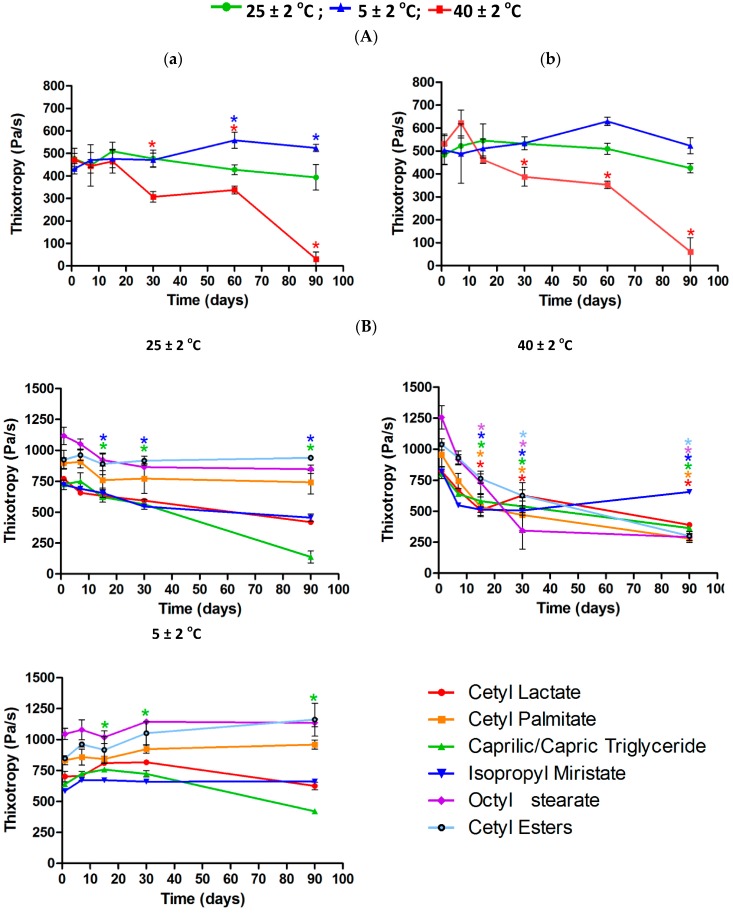
Thixotropy for 6.5 (**a**) and 7.0 (**b**) formulations (*n* = 3) without (**A**) or with (**B**) fatty esters.

Although they showed some statistically significant changes the formulations 6.5 and 7.0 without (Figure 10A) or with fatty esters (Figure 10B) stored at room temperature 25 ± 2 °C and 5 ± 2 °C remained stable and had better thixotropic profile than that kept at 40 ± 2 °C, which displayed statistically significant changes from the thirtieth day of storage.

The thixotropy can be quantitatively represented by the hysteresis area formed between the ascending and descending curves from a rheogram and it is evident when the upward curve is positioned above the down slope, with reopexia being the contrary case [20]. The degree of thixotropy for cosmetic products provides information about the capability and time that the product takes to return to its structure after skin application. Thixotropic behavior is useful for both pharmaceutical and cosmetics technology, because products for topical application that show this feature become more fluid, facilitating the spreadability and the viscosity is totally or partially recovered at the end of application, thus preventing the product from flowing [21].

## 3. Materials and Methods

### 3.1. Ingredients Used

Aqueous Phase: purified water. Oil phase: sunflower oil (INCI name: Helianthus annus oil; Lipo do Brasil, São Paulo, Brazil); mineral oil (INCI name: mineral oil, Labsynth, São Paulo, Brazil). Surfactants: Crodafos^®^ CES (INCI name: cetearyl alcohol and dicetyl phosphate and ceteth-10 phosphate, Croda do Brazil (Campinas, SP, Brazil)) named SEB I; and Dermabase^®^ Vegetal (INCI name: cetearyl alcohol (and) glyceryl stearate (and) PEG-2 stearate (and) stearic acid (and) ceteth-10 (and) polysorbate 60 (and) Theobroma grandiflorum seed butter (and) Helianthus annuus seed oil and cetyl palmitate, (Croda)) named SEB II [14]. Additives: all fatty esters (C_12–15_ alkyl benzoate; cetyl lactate; cetyl palmitate; caprylic/capric triglyceride; lauryl lactate; cetyl esters; octyl stearate; isopropyl myristate; ethylhexyl palmitate; PPG-3 benzyl ether myristate) were donated by Croda:

### 3.2. Determination of in Vitro Antioxidant Activity by H^+^ Donor Activity to DPPH^•^

Determination of the antioxidant activity of sunflower oil was performed by measuring the hydrogen ion donor activity to the stable 2,2-diphenyl-1-picrylhydrazyl radical (DPPH^•^). From the percentages of inhibition IC_50_ values were calculated for stable DPPH^•^ by the GraphPad Prism software. The IC_50_ refers to the concentration of pure oil which produces a 50% inhibition of DPPH^•^ [22,23].

### 3.3. Development of the Emulsion

#### 3.3.1. Ternary Diagram

The formulations were developed according to the ternary diagram method. The diagram was obtained using initial concentrations that increased in 10.0% *w*/*w* steps in order to obtain O/W emulsions. Only the region of the phase diagram with a high concentration of water was used, thereby giving six formulations for each proposed system. The diagram was then repeated in this region using 5.0% *w*/*w* concentration steps of the formulation components [24].

#### 3.3.2. Method

The emulsions were prepared by the Emulsion Phase Inversion (EPI) method. The aqueous and oil phases were heated separately at 75 ± 2 °C. Then, the aqueous phase was slowly poured over to the oily phases containing surfactants and keeping under stirring (600 rpm) (Mod-Fisaton mechanical stirrer, (Fisaton, São Paulo, Brazil) until room temperature (25 ± 2 °C) was reached.

#### 3.3.3. Macroscopic Analysis

The analysis of the samples were performed twenty-four hours after preparation (intrinsic stability) [25] and organoleptic properties and homogeneity of the formulations were observed, identifying possible signs of instability such as coalescence and creaming [21].

#### 3.3.4. Microscopic Analysis

The samples were directly observed under the microscope using normal and polarized light (BX 50 microscope, Olympus, Melville, NY, USA). Only emulsions with anisotropy were selected for further study. The microscopic appearance of anisotropic structures was used for the characterization of the liquid-crystalline phase [26].

### 3.4. Preliminary Stability

Macroscopically stable emulsions after twenty-four hours of preparation were subjected to the following preliminary stability tests: centrifugation, thermal stress, pH and conductivity analysis [17]. All tests were performed in triplicate.

#### 3.4.1. Centrifugation Test

The assay was performed under the following conditions: room temperature (24 ± 2 °C) and speeds of 1000, 2500 and 3500 rpm (Excelsa Baby II centrifuge, Fanem Ltd., São Paulo, Brazil) maintaining samples at each speed value for 15 min [27].

#### 3.4.2. Thermal Stress

The emulsions were subjected to thermal stress in a thermostated bath at a temperature range of 40 ± 2 to 80 ± 2 °C, increasing in 5 °C the temperature. The samples were held at each temperature for 30 min and then evaluated macroscopically [28]. For this test, the nomenclature proposed by Ribeiro *et al.* [27] was used to classify the samples: N = Normal; without change; LM = Slightly Modified; M = Modified; IM = Heavily Modified.

#### 3.4.3. pH Evaluation

The pH value was measured by inserting the electrode (Analion-Mod. PM608 pH meter, Analion, Ribeirão Preto, SP, Brazil) in the sample solution [29].

#### 3.4.4. Electrical Conductivity Evaluation

The conductivity) of the emulsions was evaluated inserting the electrode Tecnopon (Tecnopon, Piracicaba, SP, Brazil) directly into the samples at 24.0 ± 2.0 °C [29,30].

### 3.5. Extended Stability

The samples considered stable in the preliminary tests were subjected to accelerated stability testing. Emulsions were placed in clear plastic vials and kept under different storage conditions: (25 ± 2 °C); (4 ± 2 °C); (40 ± 2 °C) [27,31]. The samples were analyzed on the 1st (24 h), 7th, 15th, 30th, 60th and 90th days. The tests used to evaluate the prolonged stability were: macroscopic observation, polarized light microscopy, determination of pH, conductivity and rheology.

#### 3.5.1. Viscosity and Rheology

The viscosity and rheological properties of emulsions were studied on a rotational rheometer (Model R/S Plus Rheometer, (Brookfield, Middleboro, MA, USA) with cone-plate configuration (spindle C 50^1^, d = 50 mm, θ = 1°), operated by the Rheo 2000 V2.8 software (Brookfield, Middleboro, MA, USA). The rheological parameters were determined at 25.0 ± 0.1 °C. The gap between the cone and the plate was 0.05 mm. The input parameters used to construct the ascending and descending curves of shear stress (Pa) and the viscosity (η) are listed in Table 4.

The stability test results were studied using statistical analysis software (GraphPad Prism 5.0) to detect significant differences. One-away ANOVA analysis of variance followed by post-test Tukey multiple comparisons were used. Statistically significant differences were 95% (*p* < 0.05).

## 4. Conclusions

Natural sunflower oil showed antioxidant activity after heating the oil phase, allowing its use in the emulsification process. Emulsion systems consisting of natural sunflower oil + mineral oil and anionic and nonionic surfactants were stable and showed lamellar structures during accelerated stability tests. All formulations showed non-Newtonian flow, pseudoplastic and thixotropic behavior. The carbon chains of some fatty esters can influence the formation and maintenance of liquid crystals and do not interfere with the pH, electrical conductivity values and rheological behavior.

## Figures and Tables

**Table 1:** Composition (% *w*/*w*) of derivatives of formula 36.

Formula	Surfactant		Oil				Purified Water
	SEB I	SEB II	Total	Sunflower	Mineral	Total	
I	7.5	2.5	10.0	11.25	3.75	15.0	75.0
II	3.75	1.25	5.0	11.25	3.75	15.0	80.0
***III***	***3.75***	***1.25***	***5.0***	***7.5***	***2.5***	***10.0***	***85.0***
IV	7.5	2.5	10.0	3.75	1.25	5.0	85.0
V	11.25	3.75	15.0	3.75	1.25	5.0	80.0
VI	11.25	3.75	15.0	7.5	2.5	10.0	75.0

**Table 2:** Details of concentrations (% *w*/*w*) of derivatives of formula 36 (after 36 III): surfactant–oils (7.5:2.5 sunflower–mineral oil)-purified water.

Formula	Surfactant		Purified Water
	SEB I	SEB II	
0.5	0.38	0.12	89.5
1.0	0.75	0.25	89.0
1.5	1.125	0.375	88.5
2.0	1.5	0.5	88.0
2.5	1.38	0.62	87.5
3.0	2.25	0.75	87.0
3.5	2.63	0.82	86.5
4.0	3.0	1.0	86.0
4.5	3.38	1.12	85.5
5.0	3.75	1.25	85.0
5.5	4.13	1.37	84.5
6.0	4.5	1.5	84.0
6.5	4.88	1.62	83.5
7.0	5.25	1.75	83.0
7.5	5.63	1.87	82.5
8.0	6.0	2.0	82.0
8.5	6.38	2.12	81.5
9.0	6.75	2.25	81.0
9.5	7.13	2.37	80.5
10.0	7.25	2.75	80.0

**Table 3:** Results for preliminary stability tests of emulsions.

Formulas	Centrifugation Cycles			Thermal Stress (±2 °C)			
	I	II	III	55	60	65	70
5.5	N	N	N	N	LM	M	M
6.0	N	N	N	N	N	LM	M
**6.5**	**N**	**N**	**N**	**N**	**N**	LM	M
**7.0**	**N**	**N**	**N**	**N**	**N**	LM	M

Legend: N: Normal; LM: Slightly modified; M: Modified.

**Table 4:** Input parameters used to construct the shear gradient and viscosity ascent and descent curves.

Curve	Shear Rate		Steps (s)	Length Step (s)	Total Time (s)
	Initial (1/s)	Final (1/s)			
Ascent	10	100	10	6	60
Descent	100	10	10	6	60

The graphs relate obtained values of shear (Pa) and the viscosity (η) with the shearing rate (1/s) stress. Differential viscosity values (calculated shear rate = 100 s^−1^), flow index (exponent yield) consistency index (plastic viscosity) and thixotropy (hysteresis area) were obtained. Note: Unit 1 D/cm^2^ shear stress = 0.1 Pa; Unit 1cP viscosity = 0.001 Pas.
